# Krueppel-Like Factor 4 Expression in Phagocytes Regulates Early Inflammatory Response and Disease Severity in Pneumococcal Pneumonia

**DOI:** 10.3389/fimmu.2021.726135

**Published:** 2021-09-13

**Authors:** Toni Herta, Aritra Bhattacharyya, Maciej Rosolowski, Claudia Conrad, Corinne Gurtner, Achim D. Gruber, Peter Ahnert, Birgitt Gutbier, Doris Frey, Norbert Suttorp, Stefan Hippenstiel, Janine Zahlten

**Affiliations:** ^1^Department of Infectious Diseases and Respiratory Medicine, Charité – Universitätsmedizin Berlin, Berlin, Germany; ^2^Institute for Medical Informatics, Statistics and Epidemiology (IMISE), University of Leipzig, Leipzig, Germany; ^3^Department of Veterinary Pathology, Freie Universität Berlin, Berlin, Germany

**Keywords:** Krueppel-like factor 4, *Streptococcus pneumoniae*, community-acquired pneumonia, innate immunity, myeloid cells

## Abstract

The transcription factor Krueppel-like factor (KLF) 4 fosters the pro-inflammatory immune response in macrophages and polymorphonuclear neutrophils (PMNs) when stimulated with *Streptococcus pneumoniae*, the main causative pathogen of community-acquired pneumonia (CAP). Here, we investigated the impact of KLF4 expression in myeloid cells such as macrophages and PMNs on inflammatory response and disease severity in a pneumococcal pneumonia mouse model and in patients admitted to hospital with CAP. We found that mice with a myeloid-specific knockout of KLF4 mount an insufficient early immune response with reduced levels of pro-inflammatory cytokines and increased levels of the anti-inflammatory cytokine interleukin (IL) 10 in bronchoalveolar lavage fluid and plasma and an impaired bacterial clearance from the lungs 24 hours after infection with *S. pneumoniae*. This results in higher rates of bacteremia, increased lung tissue damage, more severe symptoms of infection and reduced survival. Higher KLF4 gene expression levels in the peripheral blood of patients with CAP at hospital admission correlate with a favourable clinical presentation (lower sequential organ failure assessment (SOFA) score), lower serum levels of IL-10 at admission, shorter hospital stay and lower mortality or requirement of intensive care unit treatment within 28 days after admission. Thus, KLF4 in myeloid cells such as macrophages and PMNs is an important regulator of the early pro-inflammatory immune response and, therefore, a potentially interesting target for therapeutic interventions in pneumococcal pneumonia.

## Introduction

*Streptococcus pneumoniae* (*S. pneumoniae*) is the most common causative pathogen of community-acquired pneumonia (CAP), an infectious disease which causes 1.2 million deaths per year worldwide (1), despite antibiotics and vaccination. The emergence of multidrug-resistant *S. pneumoniae* strains has increased the mortality and morbidity associated with pneumococcal pneumonia, while available vaccines such as the common 23-valent polysaccharide vaccine provide insufficient protection (2–4). Thus, the development of new therapeutic strategies is needed which necessitates the understanding of the underlying molecular mechanisms.

The innate immune system of the lungs is key to eliminate invading pneumococci (5, 6). Phagocytes such as polymorphonuclear neutrophils (PMNs) and macrophages are vital effectors of the cellular arm of the innate immune response of the lungs. Following the concept of the mononuclear phagocyte system, both cell types are formed from myeloid precursors in the bone marrow, circulate as quiescent PMNs or monocytes in the blood and are recruited to the lungs in response to inflammatory stimuli where they transform into activated effector cells (7–9). In addition, a subset of macrophages, termed tissue-resident macrophages, is probably recruited to the lungs before birth and gradually turns over locally throughout adult life, independently of circulating precursors (7, 10). These cells likely function as local immune sentinels (10). After recognition of bacterial components, activated PMNs and macrophages eliminate pathogens such as pneumococci by various mechanisms (e.g. phagocytosis, release of antimicrobial compounds, spanning of extracellular traps), and orchestrate the immune response *via* production of cytokines and antigen presentation to effector cells of the adaptive immune system (11, 12). Transcription factors are central control and regulatory elements of PMN and macrophage activation, and therefore potential targets for therapeutic interventions by programming cell function to a more efficient antimicrobial response. We previously showed that the recognition of *S. pneumoniae* induces the expression of the transcription factor Krueppel-like factor (KLF) 4 (HGNC:6348) in murine and human blood-derived PMNs and murine bone marrow (BM)-derived macrophages *in vitro* (13, 14). KLF4 activated pro-inflammatory and inhibited anti-inflammatory signaling pathways in both cell types, as reflected by increased secretion of the pro-inflammatory cytokines tumor necrosis factor alpha (TNF-α) and keratinocyte chemoattractant (KC) in PMNs and interleukin 1 beta (IL-1β), IL-6 and TNF-α in macrophages and decreased secretion of the anti-inflammatory cytokine IL-10 in both cell types after induction of KLF4 in response to *S. pneumoniae* stimulation (13, 14). In PMNs, the expression of KLF4 might enhance pneumococcal killing (13). To further validate KLF4 in phagocytes as potential target for therapeutic interventions to promote pneumococcal elimination, *in vivo* explorations are needed. Here, we investigated the impact of KLF4 expression in myeloid cells such as phagocytes on disease severity and regulation of the inflammatory response in a pneumococcal pneumonia mouse model. Furthermore, we correlated the KLF4 gene expression level in the peripheral blood of adult patients admitted to hospital with CAP with disease severity, anti-inflammatory IL-10 serum levels and further hospital course.

## Materials and Methods

### Mice

Floxed KLF4 mice with C57BL/6J background (ERT-Cre^+/-^/KLF4^loxP/loxP^ and ERT-Cre^-/-^/KLF4^loxP/loxP^) were kindly provided by Gary K. Owen (Department of Molecular Physiology and Biological Physics, University of Virginia, Charlottesville) and mated with B6.129P2-Lyz2tm1(Cre) Ifo mice (Charles River, Sulzfeld, Germany) to generate myeloid KLF4 knockout (KO) mice (C57BL/6 LyzMCre^+/+^/KLF4^loxP/loxP^, referred to as mKLF4 KO) and KLF4 wildtype (WT) mice (C57BL/6 LyzMCre^-/-^/KLF4^loxP/loxP^, referred to as mKLF4 WT). Experiments were performed using female mice, aged 8-11 weeks, kept in individually ventilated cages. For genotyping, murine ear tissue was lysed in Tris-EDTA-SDS buffer with 10 mg/ml Proteinase K (Sigma-Aldrich, St. Louis, MO), and PCR was performed using DreamTaq DNA Polymerase (Thermo Scientific, Waltham, MA). The primer sequences used for genotyping are listed in **Supplementary Table 2**.

### Murine Pneumococcal Pneumonia Experiments

Pneumococcal pneumonia was induced as described previously (15). In short, *S. pneumoniae* serotype 3 strain NCTC 7978 was grown to midlog-phase. Mice were anesthetized with intraperitoneal injection of ketamine and xylazine (Panpharma, Luitré, France) and 20 µl bacterial suspension in PBS (∼5x10^4^ or ∼5x10^5^ colony forming units, CFUs) or 20 µl PBS was inoculated transnasally. The mice were kept in 12 hours light/dark cycle and monitored every 12 hours for maximal 10 days. After 10 days or when level of pain reached the humane endpoint of infection (16), mice were sacrificed. Clinical symptoms of infection (breathing quality, reaction to external stimuli, raised fur) were captured as deviation from uninfected mice (= score of 0) using a semiquantitative scoring. Bronchoalveolare lavage (BAL) was performed as described previously (17), and venous blood was sampled from the *vena cava caudalis* in K_2_EDTA tubes (Sarstedt, Nümbrecht, Germany) for CFU determination or for centrifugation (10 minutes, 2000 g, 4°C) to obtain serum. CFUs were determined from serial dilutions of lung and spleen homogenates or blood, plated on Columbia agar plates with 5% sheep blood (Becton Dickinson, Franklin Lakes, NJ) and incubated for 16 hours at 37°C before colony count. Cytokines were quantified in BAL fluid (BALF) and serum using Enzyme-linked Immunosorbent Assay (ELISA) kits: TNF-α, IL-1β, IL-10 (eBioscience, San Diego, CA) and KC (R&D systems, Minneapolis, MN). Mouse albumin ELISA was performed from BALF and serum using a murine albumin ELISA kit (BioMol, Hamburg, Germany).

### Murine Lung Histology

Whole lungs with trachea were removed, immersion fixed in 4% paraformaldehyde pH 7.0 (Carl Roth, Karlsruhe, Germany) for 48 hours and embedded in paraffin (Thermo Scientific, Waltham, MA). 4 µm thick whole-lung horizontal sections were stained with hematoxylin & eosin (Carl Roth, Karlsruhe, Germany) and scored in a blinded fashion by a veterinary pathologist for the following parameters: perivascular edema, pleuritis, necrosis, area of infection. For immunohistochemical detection of *S. pneumoniae* in paraffin embedded sections, antigen retrieval was performed with microwave heating (600 W) for 12 minutes in 750 ml 10 mM citric acid pH 6.0 (Merck, Darmstadt, Germany). Sections were incubated with a purified rabbit polyclonal antibody which recognizes epitopes in the capsule, the cell wall and the cytosol of *S. pneumoniae* (1:2000, kindly provided by Sven Hammerschmidt, Interfaculty Institute for Genetics and Functional Genome Research, University of Greifswald, Germany) at 4°C overnight and subsequently incubated with an alkaline phosphatase conjugated goat anti-rabbit secondary antibody (1:500, Vector, Burlingame, CA) for 30 minutes at room temperature as described in (18).

### Human Study Subjects

Human analysis performed in this study were based on clinical data and asservated biomaterials of 371 patients recruited within the multicenter observational PROGRESS (Pneumonia Research Network on Genetic Resistance and Susceptibility for the Evolution of Severe Sepsis) study (clinicaltrials.gov: NCT02782013). Study design and PROGRESS network aims are detailed in (19). In short, patients with CAP aged 18 years or over were enrolled upon admission to hospitals in Germany and Austria. CAP was defined as pulmonary infiltrate detected by chest X-ray, no hospitalization within the last 28 days, and two or more of the following characteristics: (1) fever, (2) cough, (3) purulent sputum, (4) shortness of breath or need for respiratory support, (5) rales or bronchial breathing on auscultation or dullness to percussion. Exclusion criteria are specified in **Supplementary Table 3**. Detection of pathogens relied on blood culture, culture of respiratory material (sputum, induced sputum, BAL), urinary antigen testing for *S. pneumoniae* and *Legionella pneumophila*, and rapid Influenza diagnostic tests, depending on availability.

### Clinical and Laboratory Parameters

Baseline characteristics of patients (age, gender, nursery home residency, smoking history, comorbidities), clinical and laboratory parameters upon hospital admission and information about further hospital course (length of hospital stay, mortality and intensive care unit treatment within 28 days after admission) were collected by trained study nurses and documented using standardized electronic case report forms. The sequential organ failure assessment (SOFA) score was calculated of 6 subscores evaluating specific organ functions as defined in (20): (1) respiration: Horowitz index (PaO_2_/FiO_2_); (2) central nervous system: Glasgow Coma Scale; (3) cardiovascular system: mean arterial pressure; (4) liver: bilirubin serum level; (5) coagulation: thrombocytes; (6) kidneys: creatinine serum level. IL-10 serum levels were quantified using a LUMINEX based multiplex bead array system as described in (21).

### Microarray Analysis

Whole blood was collected into PAXgene blood RNA tubes (Qiagen, Hilden, Germany) and RNA was extracted using the PAXgene blood RNA kit (PreAnalytix, Hombrechtikon, Switzerland). Total gene expression analysis was performed using the DirectHyb HumanHT-12 v4 expression bead chip (Illumina, San Diego, CA) and the HiScan™SQ system (Illumina, San Diego, CA) as detailed in (19). Quantile normalization was used to normalize results. ILMN_2137789 represented the gene expression level of KLF4.

### Statistics

Statistical analysis was performed using GraphPad Prism 6 (Graph Pad, San Diego, CA) for mouse and R version 3.6.0 [R Core Team, University of Auckland, New Zealand (22)] for human data. Results were compared using Cox-Mantel logrank test, Spearman’s rank-order correlation, Multiple t-test, Mann-Whitney U test or Kruskal-Wallis test [non-parametric one-way analysis of variance (ANOVA)] with Dunn multiple-comparisons test as specified in the figure legends. P-values < 0.05 were considered as statistically significant.

### Study Approval

All animal housing and experimental procedures complied with the Federation of European Laboratory Animal Science Association (FELASA) guidelines and recommendations for the care and use of laboratory animals. The animal procedures were approved by the local institutional (Charité – Universiätsmedizin Berlin) and governmental (Landesamt für Gesundheit und Soziales Berlin (LAGeSo), approval ID: G0028/16) authorities.

All human study procedures complied with the ethical principles of the Declaration of Helsinki and the guideline for good clinical practice of the European Medicines Agency (EMA). The study protocol was approved by the ethics committee of the University of Jena (2403-10/08) and by locally responsible ethics committees of each study center. Written informed consent was received from participants prior to inclusion in the study.

## Results

### Knockout of KLF4 in Myeloid Cells Results in Reduced Survival and More Severe Symptoms in Murine Pneumococcal Pneumonia

First, LyzMcre-mediated KLF4 knockdown efficacy in myloid cells in our mouse model was analyzed. Therefore, blood-derived PMNs and bone marrow-derived macrophages (BMMs) were isolated from myeloid KLF4 knockout (mKLF4 KO) and KLF4 wildtype (mKLF4 WT) mice and stimulated for 6 hours with capsule-deficient R6x pneumococci (multiplicity of infection (MOI) 1). We found a KLF4 knockdown efficacy of > 80% in blood-derived PMNs (13) and of ~50% in BMMs (**Supplementary Figure S1**).

**Figure 1:**
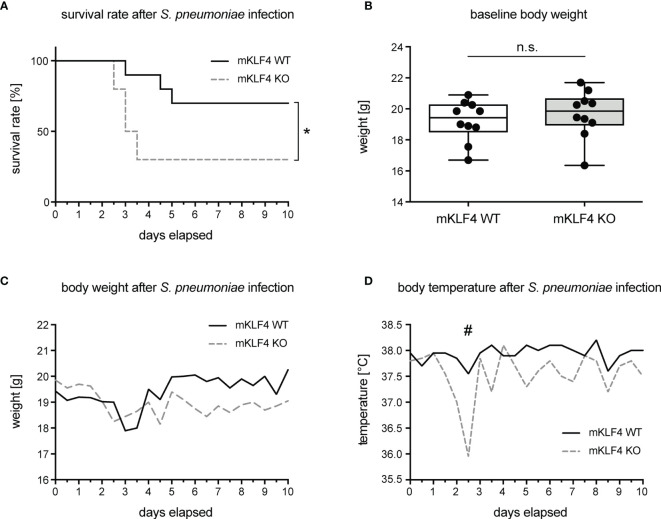
Survival rate, body weight and body temperature of myeloid KLF4 knockout and KLF4 wildtype mice after transnasal infection with *S. pneumoniae*. Myeloid KLF4 knockout (mKLF4 KO) and KLF4 wildtype (mKLF4 WT) mice were transnasally infected with 5x10^4^ CFU NCTC 7978 pneumococci and survival, body weight and body temperature were monitored every 12 hours for 10 days post infection **(A, C, D)**. Baseline body weight was determined before infection with *S. pneumoniae* **(B)**. Graphs show survival proportions of 2 groups starting with 10 mice, each **(A)**, boxplots with min to max whiskers on linear scale of 10 mice, each **(B)**, or median starting with 10 mice, each **(C, D)**. Statistics: Cox-Mantel logrank test **(A)**, Mann-Whitney U test **(B)**, or Multiple t-test **(C, D)**. *p < 0.05, ^#^p = 0.063, n.s., not significant.

To investigate the impact of KLF4 expression in myeloid cells on overall outcome and survival in pneumococcal pneumonia, mKLF4 KO and mKLF4 WT mice were transnasally infected with 5x10^4^ colony foming units (CFU) NCTC 7978 pneumococci. Survival rate, body weight and body temperature were monitored every 12 hours for 10 days post infection. Myeloid KLF4 KO mice reached the humane endpoint of infection (level of pain, determined by department-authorized veterinarian) earlier (within 2.5 to 3.5 days post infection) compared to KLF4 WT mice (within 3 to 5 days post infection) and had to be euthanized more frequently and at earlier time points than KLF4 WT mice (**Figure 1A**). There was no difference of body weight at baseline (before infection, **Figure 1B**) and in the course of infection over the period of 10 days (**Figure 1C**). Myeloid KLF4 KO mice showed a marked drop of body temperature between day 2.5 to 3 post infection compared to KLF4 WT mice (**Figure 1D**). However, body temperature did not differ in the further course of infection until day 10.

Next, myeloid KLF4 KO and KLF4 WT mice were transnasally infected with a higher dosage of NCTC 7978 pneumococci (5x10^5^ CFU) and clinical symptoms of infection (breathing quality, reaction to external stimuli, raised fur) were monitored every 12 hours for 48 hours. Myeloid KLF4 KO mice showed earlier and significant stronger symptoms of infection compared to KLF4 WT mice (**Supplementary Figure S2**). Thus, KLF4 expression in myeloid cells impacts survival rate and clinical severity of pneumococcal pneumonia in mice.

### Knockout of KLF4 in Myeloid Cells Leads to Higher Bacterial Load in Lungs and Blood, Increased Permeability of the Alveolar-Capillary Barrier and Increased Lung Tissue Damage in Murine Pneumococcal Pneumonia

**Figure 2:**
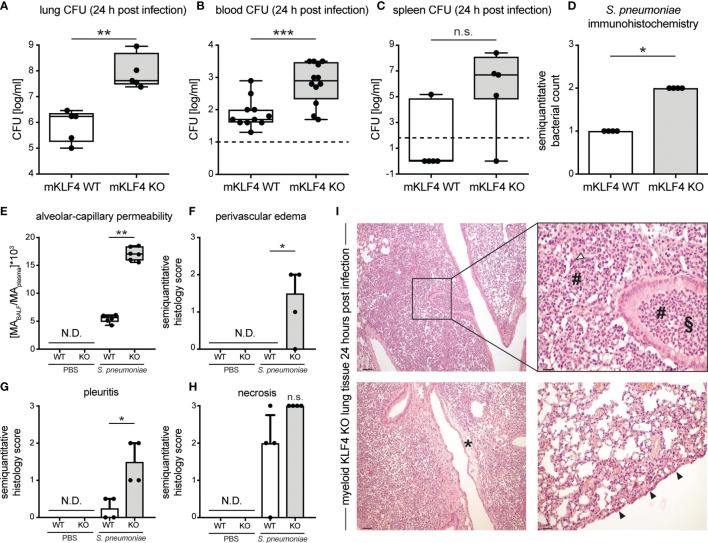
Bacterial load in lungs, blood, spleen and lung tissue damage in myeloid KLF4 knockout and KLF4 wildtype mice 24 hours after transnasal infection with *S. pneumoniae*. Myeloid KLF4 knockout (mKLF4 KO, KO) and KLF4 wildtype (mKLF4 WT, WT) mice were transnasally inoculated with 5x10^5^ CFU NCTC 7978 pneumococci **(A–I)** or PBS **(E–H)**. 24 hours after inoculation venous blood was sampled, BAL was performed and mice were sacrificed. Bacterial CFUs were determined from lung homogenates **(A)**, venous blood **(B)** and spleen homogenates **(C)** (lower limit of detection lung/spleen homogenates 50 cfu/ml, venous blood 10 cfu/ml, dotted line). Murine albumin (MA) in BALF and plasma was quantified using ELISA **(E)**. Whole lung sections were stained using a polyclonal antibody against *S. pneumoniae* **(D)** or hematoxylin & eosin **(F–I)** and semiquantitatively scored for bacterial count **(D)**, perivascular edema formation **(F)**, pleuritis formation **(G)** and necrosis formation **(H)**. Graphs show boxplots with min to max whiskers on base-10 log scale of 5 mice, each **(A, C)**, boxplots with min to max whiskers on base-10 log scale of 11 mice (mKLF4 WT) or 12 mice (mKLF4 KO) **(B)**, median with interquartile range of 4 mice, each **(D, F–H)**, or boxplots with min to max whiskers on linear scale of 6 mice, each **(E)**. **(I)** shows representative sections of lung tissue of mKLF4 KO mice 24 hours after infection with *S. pneumoniae* (hematoxylin & eosin staining). # PMN infiltrate, ∆ necrosis formation, § suppurative bronchopneumonia, * perivascular edema formation, ▲ pleuritis formation. Scale bars: 100 µm upper and lower left, 20 µm upper right, and 50 µm lower right picture. Statistics: Mann-Whitney U test **(A–E)**, or Kruskal-Wallis test with Dunn multiple-comparisons test **(F–H)**. *p < 0.05, **p < 0.01, *** < 0.001, n.s., not significant; N.D., not detectable.

Severity of pneumococcal pneumonia is associated with higher bacterial load, bacteremia, impairment of the alveolar-capillary barrier and increased lung tissue damage. To assess the impact of KLF4 expression in myeloid cells on bacterial load in the lungs and bacteremia, myeloid KLF4 KO and KLF4 WT mice were transnasally infected with 5x10^5^ CFU NCTC 7978 pneumococci. 24 hours post infection venous blood was sampled and mice were sacrificed. Lung and spleen samples were homogenized for CFU determination and lung tissue was fixed for immunohistochemistry. Myeloid KLF4 KO mice showed a significant higher bacterial load in lungs and blood and tended to have a higher bacterial load in the spleen 24 hours post infection compared to KLF4 WT mice (**Figures 2A–C**). Semiquantitative scoring of immunohistochemistry staining of lung sections with a polyclonal antibody against *S. pneumoniae* confirmed the higher amount of bacteria in the lungs of myeloid KLF4 KO mice 24 hours after infection (**Figure 2D**).

Post infection integrity of the alveolar-capillary barrier in myeloid KLF4 KO and KLF4 WT mice was assessed by determining the concentration of albumin in the bronchoalveolare lavage fluid (BALF) over that in plasma 24 hours after transnasal inoculation with PBS or 5x10^5^ CFU NCTC 7978 pneumococci. Myeloid KLF4 KO mice showed a higher alveolar-capillary permeability 24 hours post infection compared to KLF4 WT mice (**Figure 2E**). No albumin was detected in the alvealor space of PBS treated mice. In line with the increased alveolar-capillary permeability, histological scoring of the lungs 24 hours after infection with 5x10^5^ CFU NCTC 7978 pneumococci revealed a significant increase in perivascular edema and pleuritis formation and a tendency towards increased necrosis formation and area of pneumonia affected lung tissue in myeloid KLF4 KO mice in comparison with KLF4 WT mice (**Figures 2F–I** and **Supplementary Figure S3**). No histological signs of inflammation were found in the lungs of PBS treated mice. Thus, KLF4 expression in myeloid cells increases bacterial clearance, reduces bacteremia, promotes alveolar-capillary barrier integrity and reduces lung tissue damage in murine pneumococcal pneumonia.

### Knockout of KLF4 in Myeloid Cells Results in Lower TNF-α, KC and IL-1β and Higher IL-10 Cytokine Levels in Bronchoalveolar Lavage Fluid and Plasma in Murine Pneumococcal Pneumonia

**Figure 3:**
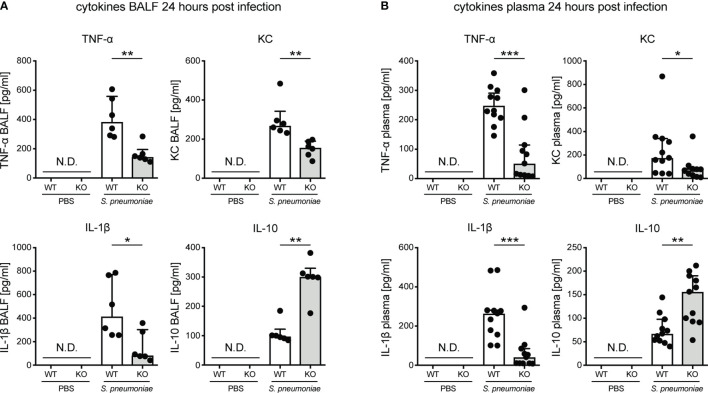
Cytokines in bronchoalveolar lavage fluid and plasma of myeloid KLF4 knockout and KLF4 wildtype mice 24 hours after transnasal infection with *S. pneumoniae*. Myeloid KLF4 knockout (KO) and KLF4 wildtype (WT) mice were transnasally inoculated with PBS or 5x10^5^ CFU NCTC 7978 pneumococci. 24 hours after inoculation venous blood was sampled and BAL was performed. TNF-α, KC, IL-1β and IL-10 was quantified in BALF **(A)** and plasma **(B)** using ELISA. Graphs show median with interquartile range of 6 mice, each **(A)**, or median with interquartile range of 11 mice, each **(B)**. Statistics: Mann-Whitney U test. *p < 0.05, **p < 0.01, ***p < 0.001. N.D., not detectable.

We previously showed that induction of KLF4 expression in myeloid cells such as PMNs and macrophages by *S. pneumoniae* leads to increased secretion of pro-inflammatory cytokines (TNF-α, KC, IL-6 and IL-1β) and reduced secretion of the anti-inflammatory cytokine IL-10 *in vitro* (13, 14). Hence, *in vivo* cytokine levels were determined in BALF and plasma in myeloid KLF4 KO and KLF4 WT mice 24 hours after transnasal inoculation with PBS or 5x10^5^ CFU NCTC 7978 pneumococci. Myeloid KLF4 KO mice showed reduced levels of TNF-α, KC and IL-1β and increased levels of IL-10 in BALF (**Figure 3A**) and plasma (**Figure 3B**) after infection with *S. pneumoniae*. No cytokine secretion was measured in PBS treated mice. Interestingly, PMN cell count in BALF did not differ between myeloid KLF4 KO and KLF4 WT mice 24 hours after infection (**Supplementary Figure S4**). Thus, in line with our *in vitro* findings, *in vivo* expression of KLF4 in myeloid cells increases the release of the pro-inflammatory cytokines TNF-α, KC and IL-1β and decreases the release of the anti-inflammatory cytokine IL-10 in murine pneumococcal pneumonia. However, decreased release of pro-inflammatory cytokines does not impair PMN recruitment to the lungs in myeloid KLF4 KO mice.

### KLF4 Gene Expression Levels in Peripheral Blood of Patients With Community-Acquired Pneumonia Correlate With SOFA Score and Serum IL-10 Cytokine Levels Upon Admission, Length of Hospital Stay and Death or Intensive Care Unit Treatment Within 28 Days After Admission

**Figure 4:**
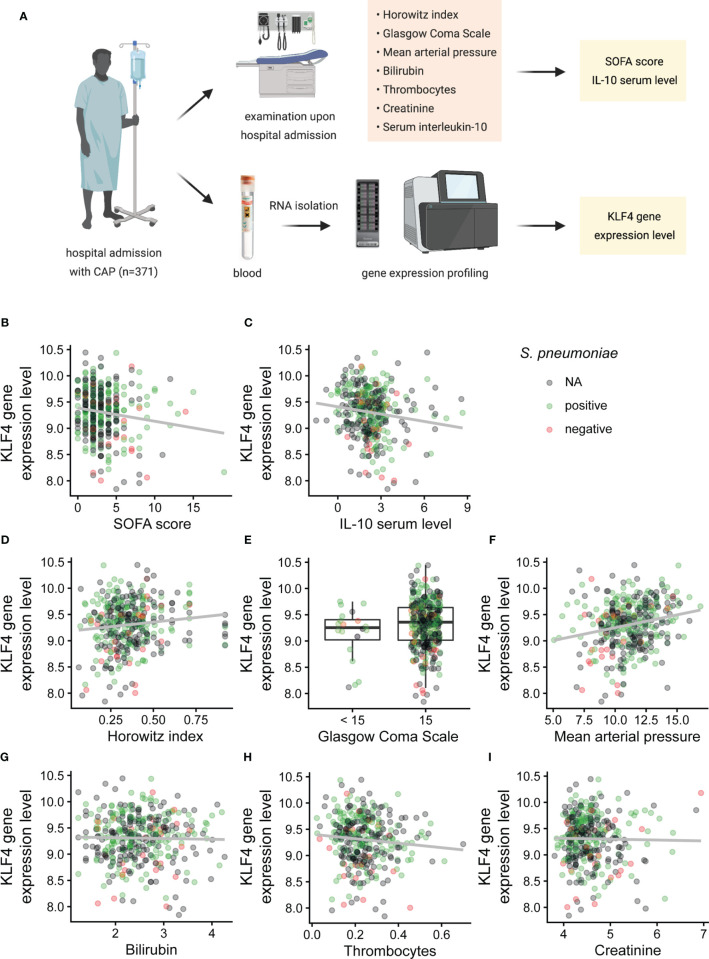
Correlation of KLF4 gene expression levels in peripheral blood with SOFA score, SOFA subscores and serum levels of IL-10 in patients with community-acquired pneumonia at admission to hospital. Horowitz index (oxygenation index, PaO_2_/FiO_2_), Glasgow Coma Scale, mean arterial pressure, total bilirubin, thrombocyte count, serum creatinine, IL-10 serum levels and KLF4 gene expression levels in peripheral blood were determined in 371 patients with community-acquired pneumonia (CAP) at presentation to hospital **(A)**. KLF4 gene expression levels (in copies per cell, vertical axis) were correlated with the SOFA score (in points) **(B)**, the IL-10 serum concentration (in pg/ml, values natural log-transformed) **(C)** and the SOFA subscores: Horowitz index (in kPa, original values divided by 100) **(D)**, Glasgow Coma Scale (in points, values split into the categories 15 points or below 15 points) **(E)**, mean arterial pressure (in kPa) **(F)**, serum bilirubin (in µmol/l, values natural log-transformed) **(G)**, thrombocyte count (in millions/µl) **(H)**, and serum creatinine (in µmol/l, values natural log-transformed) **(I)**. Green dots represent patients with positive testing for *S. pneumoniae* (blood culture, culture of respiratory material, or urinary antigen testing), red dots represent patients with negative testing for *S. pneumoniae*, and grey dots represent patients without testing for *S. pneumoniae* (N.A.). Graphs show scatter plots with trendline **(B–D, F–I)**, or boxplots with interquartile range **(E)**. Data points overlaying the boxplots are scattered horizontally. FiO_2_, fraction of oxygen in the inhaled air (in percent); PaO_2_, oxygen partial pressure in capillary or arterial blood (in mmHg).

KLF4 in human peripheral blood is mainly expressed in phagocytes (in decreasing order of frequency: monocytes, dendritic cells, PMNs, natural killer cells), as depicted in the combined gene expression consensus dataset of the Human Protein Atlas (HPA), the Genotype-Tissue Expression (GTEx) Portal and the FANTOM5 project (proteinatlas.org/ENSG00000136826-KLF4/blood) (23–28). We therefore correlated the KLF4 gene expression level in the peripheral blood of patients admitted to hospital with community-acquired pneumonia (CAP) with the sequential organ failure assessment (SOFA) score as validated scoring system for CAP severity in adult patients (29), the serum IL-10 cytokine level upon admission, the length of hospital stay and death or intensive care unit treatment within 28 days after admission (**Figure 4A**). Demographic and clinical characteristics of the patient cohort are shown in **Supplementary Table 1**. Patients with higher KLF4 gene expression had a lower SOFA score as indicator for less severe pneumonia (mainly due to better oxygenation, higher mean arterial pressure, unaltered level of consciousness) and lower anti-inflammatory IL-10 serum levels upon hospital admission (**Figures 4B–F**). However, higher KLF4 expression correlated with a lower thrombocyte count (**Figure 4H**). Liver and kidney function did not show a correlation with KLF4 expression (**Figures 4G, I**). Correlation coefficients and p-values are shown in **Table 1**. Patients with higher KLF4 gene expression levels at admission were less likely to die or to require intensive care unit treatment within 28 days after admission (regardless of the initial SOFA score), and had a shorter hospital stay (**Supplementary Figure S5**). Thus, higher KLF4 gene expression levels in peripheral blood at hospital admission correlate with a lower SOFA score and lower IL-10 serum levels and a favourable further hospital stay in adult patients with CAP.

**Table 1:** Correlation coefficients and p-values for the correlation of KLF4 gene expression levels in peripheral blood with SOFA score, SOFA subscores and serum levels of IL-10 in patients with community-acquired pneumonia at admission to hospital.

Parameter	Correlation coefficients	P-values
SOFA score	-0.111	0.032
IL-10 serum levels	-0.176	0.001
Horowitz index	0.103	0.048
Glasgow Coma Scale 15	0.179*	0.179
Mean arterial pressure	0.210	4.655e-05
Bilirubin	0.023	0.658
Thrombocytes	-0.107	0.045
Creatinine	-0.008	0.878

*difference of the mean expression of KLF4 between patients with a Glasgow Coma Scale of 15 points and below 15 points. Statistics: Spearman’s rank-order correlation for continuous measurements, or Mann-Whitney U test to compare 2 groups (Glasgow Coma Scale).

## Discussion

In the present study, we identified the transcription factor KLF4 in myeloid cells such as phagocytes as potent mediator of the early pro-inflammatory innate immune response and, therefore, as potentially interesting target for therapeutic interventions in pneumococcal pneumonia. The following observations support this finding: (i) Mice with a myeloid-specific knockout of KLF4 showed an impaired early pro-inflammatory immune response as reflected by reduced levels of pro-inflammatory cytokines (TNF-α, KC, IL-1β) and increased levels of the anti-inflammatory cytokine IL-10 in plasma and BALF and an impaired bacterial clearance from the lungs 24 hours after infection with *S. pneumoniae*. Likely as a result of the altered early immune response, we found that lung tissue damage, rates of bacteremia, clinical symptoms of infection and necessity for euthanasia due to reached level of pain was significantly higher in myeloid KLF4 knockout mice compared to the corresponding wildtypes. The early immune response to invading pathogens in the lungs is mainly mediated by macrophages and PMNs (30). The applied LyzMcre system resulted in a deletion efficiency of ~50% in mature macrophages and of > 80% in PMNs. In contrast, dentritic cells show only a partial deletion in this mouse model and no significant deletion is obtained in T and B cells (31). We previously described that stimulation of macrophages and PMNs with *S. pneumoniae* strongly induced the expression of KLF4 and, thereby, a pro-inflammatory phenotype in both cell types (13, 14). In correspondence with the present *in vivo* results, KLF4 increased the release of TNF-α and IL-1β in macrophages and of TNF-α and KC in PMNs, and reduced the release of IL-10 in both. The molecular mechanisms underlying this regulation comprise activation of target gene transcription e.g. by recruitment of histoneacetylase coactivators (32–35), repression of target gene transcription e.g. by recruitment of corepressors (36–38), and direct interaction of KLF4 with the transcriptional activity of other fundamental transcriptional regulators of the innate immune response (e.g. NF*-*κB or I*κ*Bα) (32, 33, 39). As PMN recruitment to the lungs was not reduced in myeloid KLF4 knockout mice, the impaired bacterial clearance from the lungs might partly correspond to the reduced pneumococcal killing which we observed in KLF4 knockout PMNs (13). Myeloid KLF4 knockout mice had to be euthanized early in the course of infection (within 2.5 to 3.5 days), preceded by a drop of body temperature between day 1.5 and day 3. The uneventful later course (reincrease of body temperature, stable body weight, stable survival rate until day 10) might indicate that adaptive immunity, which transpires several days post-infection (40, 41), is not impaired in myeloid KLF4 knockout mice. (ii) Higher gene expression levels of KLF4 in the peripheral blood of patients with CAP correlate with a favourable clinical presentation (lower SOFA score) upon hospital admission and a favourable further hospital course (shorter hospitalisation and lower mortality or requirement of intensive care unit treatment within 28 days after admission). KLF4 in human peripheral blood is mainly expressed in monocytes and PMNs (normalized expression (NX) values based on transcripts per kilobase million of the HPA, GTEx and FANTOM5 dataset: 15.9 in monocytes and PMNs, 4.8 in dendritic cells, 0.1 in natural killer cells). Higher bacterial load of *S. pneumoniae* at admission to hospital is associated with disease severity in pneumococcal pneumonia (42–45). Based on our findings in mice we conclude, that the expression of KLF4 in monocytes/macrophages and PMNs promotes the early pro-inflammatory immune response and, thereby, pneumococcal elimination in patients with pneumococcal pneumonia, which might result in reduced disease severity upon presentation to hospital and eventually a favourable further hospital course. Cytokines detected in peripheral venous blood of patients with severe pneumococcal pneumonia represent a systemic extension of the compartmentalized immune response in the lungs (46). Macrophages and PMNs, upon recognition of *S. pneumoniae*, are important producers of IL-10 in pneumococcal pneumonia (47, 48). In line with our *in vitro* results (13, 14) and the results in mice (expression of KLF4 reduces the release of IL-10 in macrophages, PMNs and *in vivo* in response to *S. pneumoniae* infection), we found that patients with higher gene expression levels of KLF4 in the peripheral blood had lower serum levels of IL-10. The anti-inflammatory cytokine IL-10 is a two faced cytokine in human infectious diseases (47, 49). While it attenuates exaggerated immune responses that can lead to deleterious tissue lesions, high levels of IL-10 in the early course of an infection impair the host’s ability to mount an effective immune response (49). Transnasal inoculation of *S. pneumoniae* in combination with IL-10 resulted in impaired bacterial clearance from the lungs, bacteremia and early lethality in mice (15). Serum levels of IL-10 upon admission to hospital were higher in patients with severe CAP meeting systemic inflammatory response syndrome (SIRS) criteria (50) and in those in need of intensive care unit admittance and mechanical ventilation (51) compared to patients with non-severe CAP. High serum levels of IL-10 have been associated with tachypnea (as indicator for poor oxygenation) and low systolic blood pressure in patients with CAP at admission to healthcare centers (52), which corresponds to our observations in patients with higher gene expression levels of KLF4 (who present with lower serum IL-10, better oxygenation and higher mean arterial pressure). To conclude, the expression of KLF4 in monocytes/macrophages and PMNs might reduce the release of IL-10 in patients with pneumococcal pneumonia and, thereby, strengthen the early innate immune response against *S. pneumoniae*.

Based on our results in mice and humans it is tempting to discuss a potential induction of KLF4 expression in macrophages and PMNs as therapeutic intervention to promote pneumococcal elimination. Antibiotic-independent immunostimulatory therapies to improve immunity against *S. pneumoniae* (e.g. by the use of adenovirus-mediated gene delivery) might be of interest for the prevention and the treatment of pneumococcal pneumonia (53–56). However, the induction of KLF4 expression as therapeutic intervention has to meet two important criteria: (i) Exclusive targeting of macrophages and PMNs as KLF4 expression in bronchial epithelial cells opposes the pro-inflammatory effect in phagocytes (*S. pneumoniae*-mediated induction of KLF4 expression reduces the release pro-inflammatory cytokines and increases the release of the anti-inflammatory cytokine IL-10 in bronchial epithelial cells, possibly as a safety mechanism to prevent hyperinflammation) (33, 57). (ii) Transient induction of KLF4 expression or molecular off switch as prolonged hyperinflammation exceeding the initial phase of infection likely causes deleterious collateral damage to host lung tissue (8, 58, 59).

In summary, the obtained results identify the transcription factor KLF4 in myeloid cells such as macrophages and PMNs as important regulator of the early pro-inflammatory innate immune response and, thereby, early disease severity in murine and human pneumococcal pneumonia. The results provide a rational to further investigate the induction of KLF4 expression in alveolar macrophages and PMNs as potential therapeutic intervention to improve immunity against *S. pneumoniae*.

## Data Availability Statement

The original contributions presented in the study are included in the article/**Supplementary Material**. Further inquiries can be directed to the corresponding author.

## Ethics Statement

The studies involving human participants were reviewed and approved by ethics committee of the University of Jena. The patients/participants provided their written informed consent to participate in this study. The animal study was reviewed and approved by Landesamt für Gesundheit und Soziales Berlin (LAGeSo).

## Author Contributions

JZ and SH conceived the murine part of the study, JZ and TH conceived the human part of the study. JZ, TH, and AB analyzed the data. AB, BG, and CC performed the murine *in vivo* experiments. CG and AG stained the murine lung tissue and analyzed the sections. MR and PA processed the human data. JZ, NS, and SH supervised the project. TH wrote the manuscript. TH, AB, and MR prepared the figures. All authors reviewed and edited the manuscript. The first-author position was assigned based on amount of intellectual work on this study. All authors contributed to the article and approved the submitted version.

## Funding

This work was supported by DFG SFB-TR84 grants to JZ, SH, AG, and NS (projects C2, B1, Z1b and B6), Jürgen Manchot Stiftung to TH and AB and the German Federal Ministry of Education and Research (BMBF) within the framework of the e:Med research and funding concept (project SYMAPATH, grant number 01ZX1906B) and the German Center fur Lung Research (PROGRESS, grant numbers 82DZU19A2 und 82DZLJ19B2), MR, PA, and NS.

## Conflict of Interest

The authors declare that the research was conducted in the absence of any commercial or financial relationships that could be construed as a potential conflict of interest.

## Publisher’s Note

All claims expressed in this article are solely those of the authors and do not necessarily represent those of their affiliated organizations, or those of the publisher, the editors and the reviewers. Any product that may be evaluated in this article, or claim that may be made by its manufacturer, is not guaranteed or endorsed by the publisher.

## Abbreviations

BAL, bronchoalveolare lavage; BALF, BAL fluid; BM, bone marrow; CAP, community-acquired pneumonia; CFU, colony forming unit; ELISA, Enzyme-linked Immunosorbent Assay; IL, interleukin; KC, keratinocyte chemoattractant; KLF, Krueppel-like factor; PMN, polymorphonuclear neutrophil; MA, murine albumin; mKLF4 KO, myeloid KLF4 knockout; mKLF4 WT, KLF4 wildtype; SOFA, sequential organ failure assessment; *S. pneumoniae*, *Streptococcus pneumoniae*; TNF-α, tumor necrosis factor alpha.
