# Supplementary material for: Krueppel-Like Factor 4 Expression in Phagocytes Regulates Early Inflammatory Response and Disease Severity in Pneumococcal Pneumonia
Source: Front Immunol. 2021 Sep 13;12:726135. doi: 10.3389/fimmu.2021.726135 (PMC8473698; doi:10.3389/fimmu.2021.726135)
Supplement: Supplementary file 1 [file DataSheet_1.docx]

Supplementary Material

Table of content

Supplementary Methods……………………………………………………………………….……....2

Supplementary Figure S1……………..……………………..……………………………….……......3

Supplementary Figure S2………………..…………………..………………………………………...4

Supplementary Figure S3………………..…………………..………………………………………...6

Supplementary Figure S4………………..…………………..………………………………………...7

Supplementary Figure S5………………..…………………..………………………………………...8

Supplementary Table 1…………...…………………………………………………………................9

Supplementary Table 2………………………………...……………………………………………...11

Supplementary Table 3………………………………...……………………………………………...12

**Supplementary Methods**

**Isolation and stimulation of bone marrow-derived macrophages (BMMs)**

For the isolation of bone marrow-derived cells (BMCs) from femurs and tibiae, myeloid KLF4 knockout (mKLF4 KO) and KLF4 wildtype (mKLF4 WT) mice were anaesthetized with intraperitoneal injection of ketamine and xylazine (Panpharma, France). Bone marrow-derived macrophages (BMMs) were differentiated from BMCs by culturing in RPMI 1640 medium containing 30% L cell supernatant (catalog #3368, Cell Biologics, USA) and 20% fetal calf serum (FCS). One day prior to infection medium was replaced by RPMI 1640 containing 15% L cell supernatant and 10% FCS. Mature mKLF4 WT and mKLF4 KO BMMs were stimulated for 6 hours with 1x10^6^ colony forming units (CFU)/ml unencapsulated *Streptococcus pneumoniae* D39 mutant R6x (multiplicity of infection (MOI) 1).

**Western blot**

Sample preparation and Western blot analysis was performed as described in Bhattacharyya A et al. (Front Microbiol 2021). Cells were lysed in NP40-based lysis buffer. 40 micrograms of total protein was subjected to SDS-PAGE (10% gels) and transferred to Hybond-ECL membranes (GE Healthcare, USA). Blocking of membranes was performed with Odyssey blocking buffer (LI-COR Biosciences, USA) for 2 hours at room temperature and incubated with primary antibodies against murine KLF4 (catalog #sc-20691, Santa Cruz Biotechnology, USA) or β-Actin (catalog #sc-130656, Santa Cruz Biotechnology, USA) in a dilution of 1:1000 (each) overnight at 4°C. Membranes were washed with PBST (1x PBS + 0.01% Tween-20) and incubated with the secondary antibodies anti-rabbit Cy5.5 (1:2000) or anti-goat IRDye800 (1:2000) (Rockland, USA) for 1 hour at room temperature. Protein levels were detected and quantified using LI-COR Odyssey 2.0 (LI-COR Biosciences, USA).

**Analysis of PMN recruitment to bronchoalveolar lavage fluid (BALF)**

Myeloid KLF4 knockout (mKLF4 KO) and KLF4 wildtype (mKLF4 WT) mice were transnasally inoculated with 5x10^5^ CFU NCTC 7978 pneumococci or PBS. 24 hours post infection bronchoalveolar lavage (BAL) was performed. 10 µl of surface block (aCD16/32 in FACS buffer (0.2% BSA in PBS)) was added to 100 µl of BAL fluid (BALF) in FACS tubes and incubated for 5 minutes on ice. Thereafter, the surface staining antibody mixture for PMN flow cytometry identification (Ly-6G (1A8) PerCP-Cy5.5 (Biolegend, USA) and CD11b (M1/70) PE-Cy7 (eBioscience, UK)) was added and incubated for 30 minutes on ice in the dark. Cells were then washed with PBS and centrifuged for 5 minutes at 425 g (4 °C). Supernatant was discarded and cells were fixed with 1% paraformaldehyde in PBS (overnight at 4 °C). After fixation, tubes were filled with FACS buffer and centrifuged (425 g, 5 minutes, 4 °C). Supernatant was discarded and cells were resuspended in 100 µl FACS buffer for flow cytometry (BD FACSCanto-II, BD Biosciences, USA). PMN cell count was quantified using FlowJo version 10.4.2 (FlowJo, USA).

**
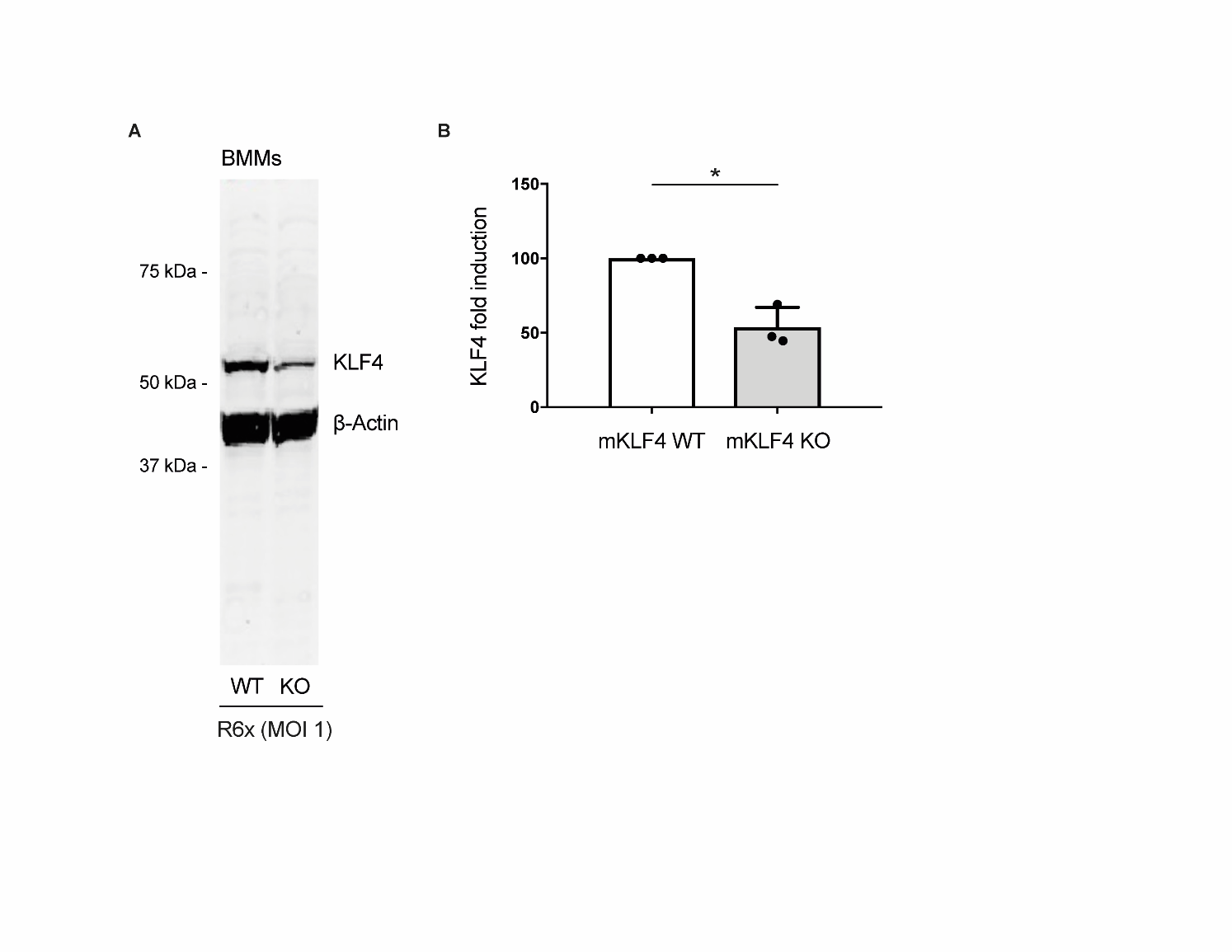
**

**Supplementary Figure S1: LyzMcre-mediated KLF4 knockout in bone marrow-derived macrophages (BMMs).** Bone marrow-derived macrophages (BMMs) were isolated from KLF4 wildtype (mKLF4 WT, WT) and myeloid KLF4 knockout (mKLF4 KO, KO) mice and stimulated with R6x pneumococci (multiplicity of infection (MOI) 1 for 6 hours). Cell lysates were collected after stimulation and analyzed for KLF4 expression using Western blotting. ß-Actin confirmed equal protein loading (A). The densitometries of the KLF4 and the ß-Actin bands were quantified using Odyssey 2.0 infrared imaging system. The ratios of the KLF4 and ß-Actin densitometries were calculated and are shown as the fold change of induction to KLF4 WT BMMs. Quantification shows the mean with standard deviation of three independent experiments (B). Statistics: Kruskal-Wallis test with Dunn multiple-comparisons test. * p<0.05.


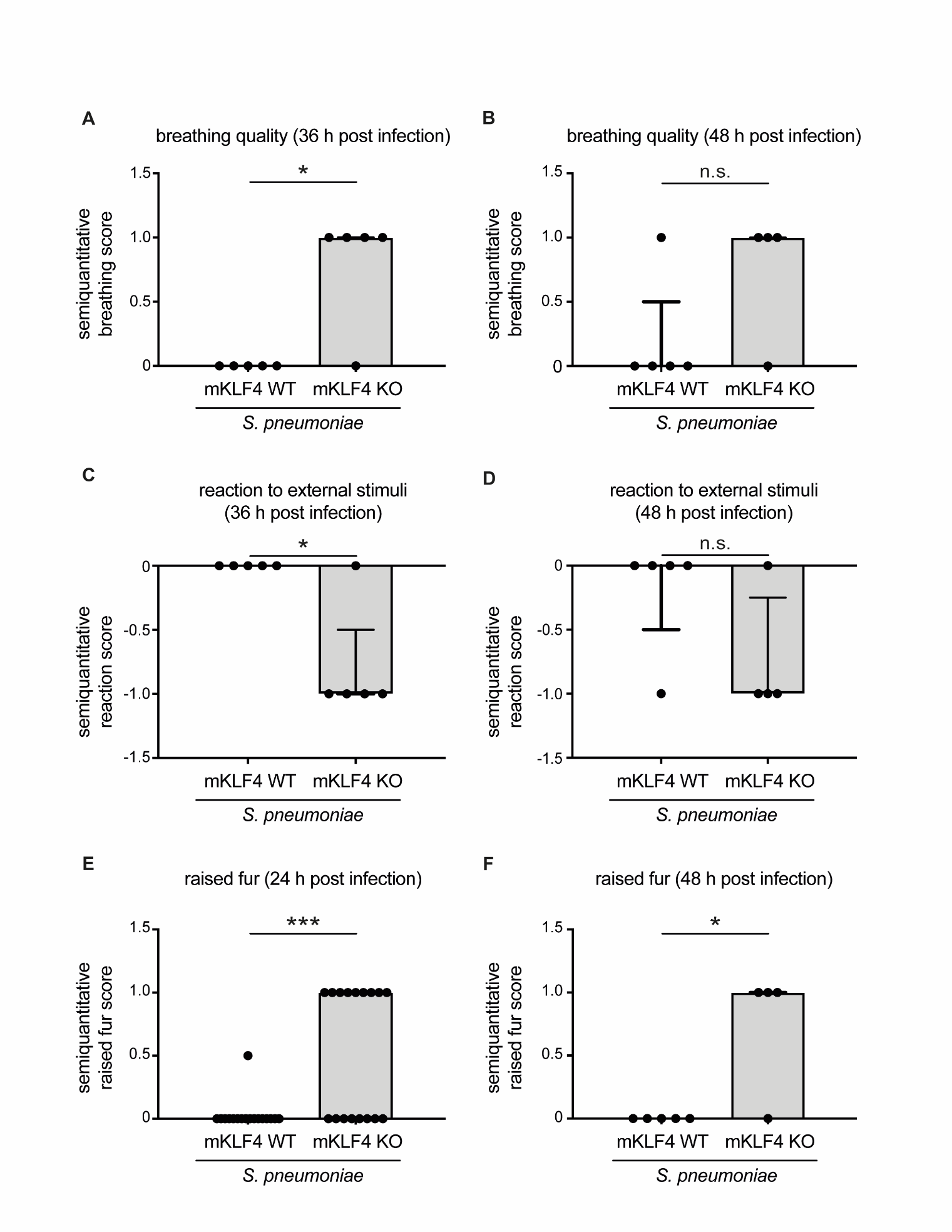


**Supplementary Figure S2: Clinical symptoms of infection in myeloid KLF4 knockout and KLF4 wildtype mice after transnasal infection with *S. pneumoniae*.** Myeloid KLF4 knockout (mKLF4 KO, grey bars) and KLF4 wildtype (mKLF4 WT, white bars) mice were transnasally infected with 5x10^5^ CFU NCTC 7978 pneumococci and clinical symptoms of infection (breathing quality, reaction to external stimuli, raised fur) were monitored every 12 hours for 48 hours (semiquantitative scoring). While scoring in mKLF4 WT mice was comparable to uninfected mice (= score 0), mKLF4 KO mice showed a strongly impaired breathing quality 36 hours post infection (A), a slower reaction to external stimuli 36 hours post infection (B), a higher incidence of raised fur 24 and 48 hours post infection (E/F) and a tendency towards a more impaired breathing quality (B) and a slower reaction to external stimuli (D) 48 hours post infection compared to mKLF4 WT mice. Graphs show median with interquartile range of 5 mice, each (A, C), 4 mKLF4 KO and 5 mKLF4 WT mice (B, D, F), or 17 mice, each (E). Statistics: Mann-Whitney U test. * p<0.05, *** p<0.001, n.s., not significant.


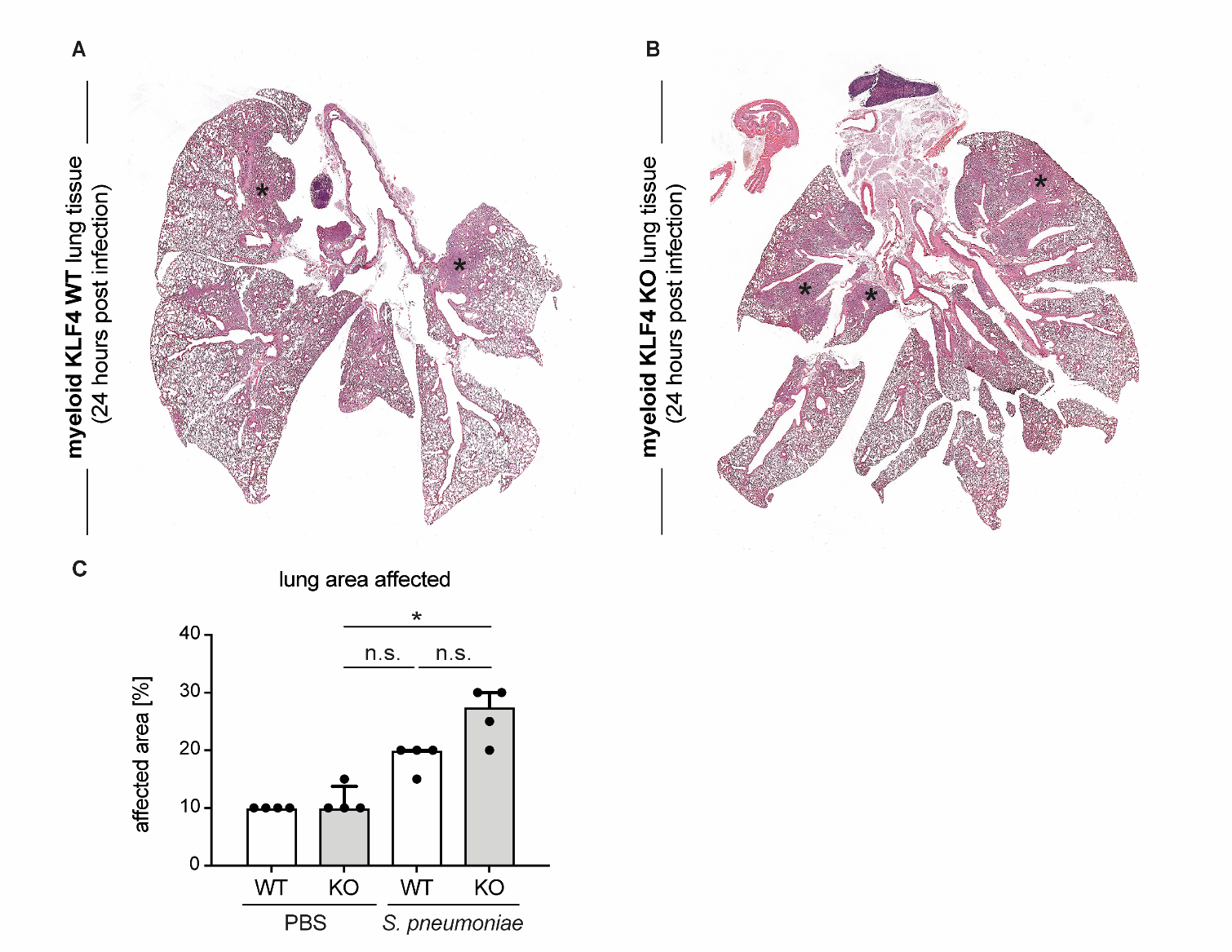


**Supplementary Figure S3: Area of pneumonia affected lung tissue in myeloid KLF4 knockout and KLF4 wildtype mice after transnasal infection with *S. pneumoniae*.** Myeloid KLF4 knockout (KO, grey bars) and KLF4 wildtype (WT, white bars) mice were transnasally inoculated with PBS or 5x10^5^ CFU NCTC 7978 pneumococci and sacrificed 24 hours post infection. Hematoxylin & eosin stained whole lung sections with trachea were blindly scored by a veterinary pathologist for lung area affected (*). mKLF4 KO mice showed a tendency towards increased area of pneumonia affected lung tissue compared to mKLF4 WT mice after infection with *S. pneumoniae* (C). Shown here is a representative section of lung tissue 24 hours post infection of mKLF4 WT (A) and mKLF4 KO (B) mice. Graph shows median with interquartile range of 4 mice, each (C). Statistics: Kruskal-Wallis test with Dunn multiple-comparisons test. * p<0.05, n.s., not significant.

**
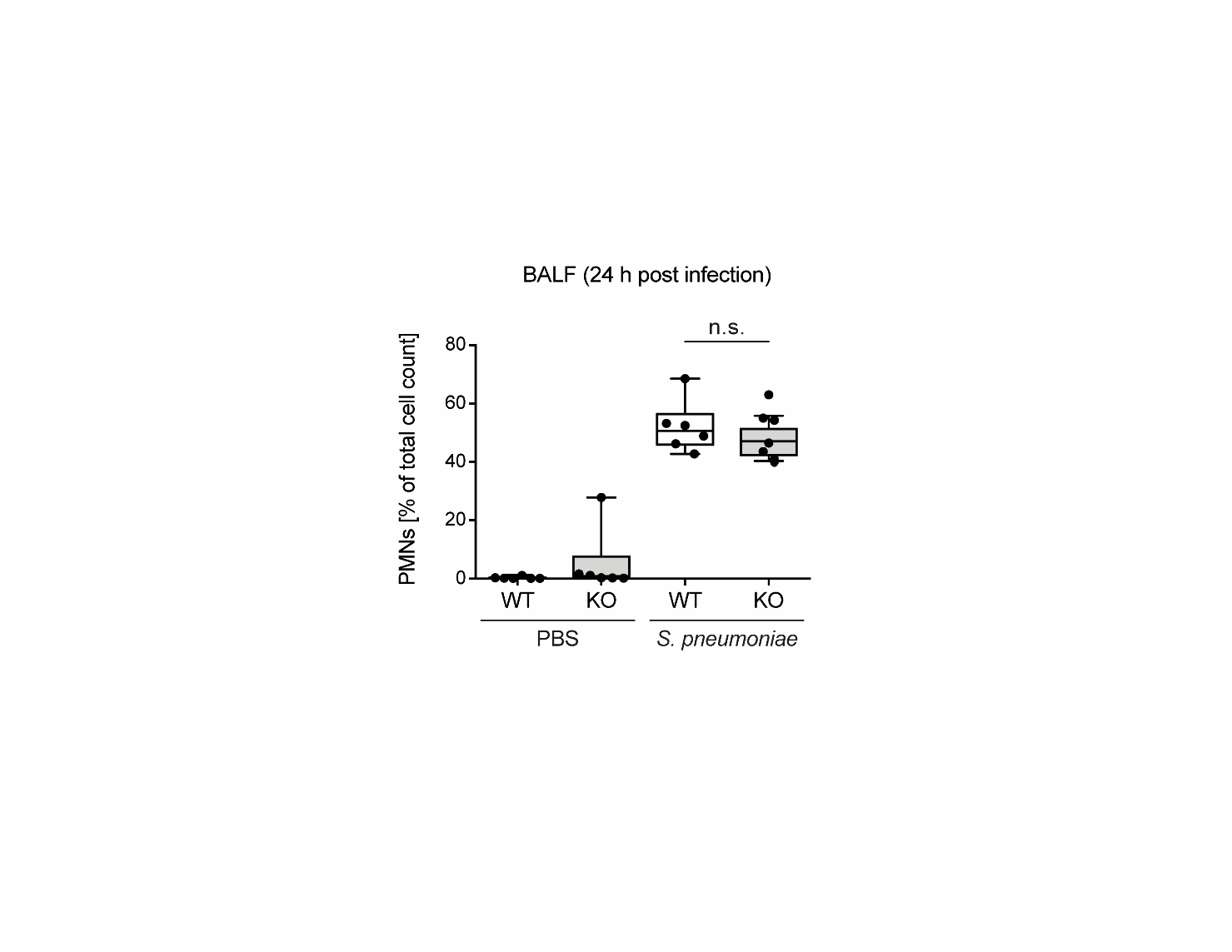
**

**Supplementary Figure S4: PMNs in bronchoalveolar lavage fluid (BALF) in myeloid KLF4 knockout and KLF4 wildtype mice 24 hours after transnasal infection with *S. pneumoniae*.** Myeloid KLF4 knockout (KO) and KLF4 wildtype (WT) mice were transnasally inoculated with PBS or 5x10^5^ CFU NCTC 7978 pneumococci. 24 hours post infection bronchoalveolar lavage (BAL) was performed. PMNs were quantified as percent of total cell count in BAL fluid (BALF). Graph shows boxplots with min to max whiskers on linear scale of 6 mice, each (B). Statistics: Kruskal-Wallis test with Dunn multiple-comparisons test. n.s., not significant.


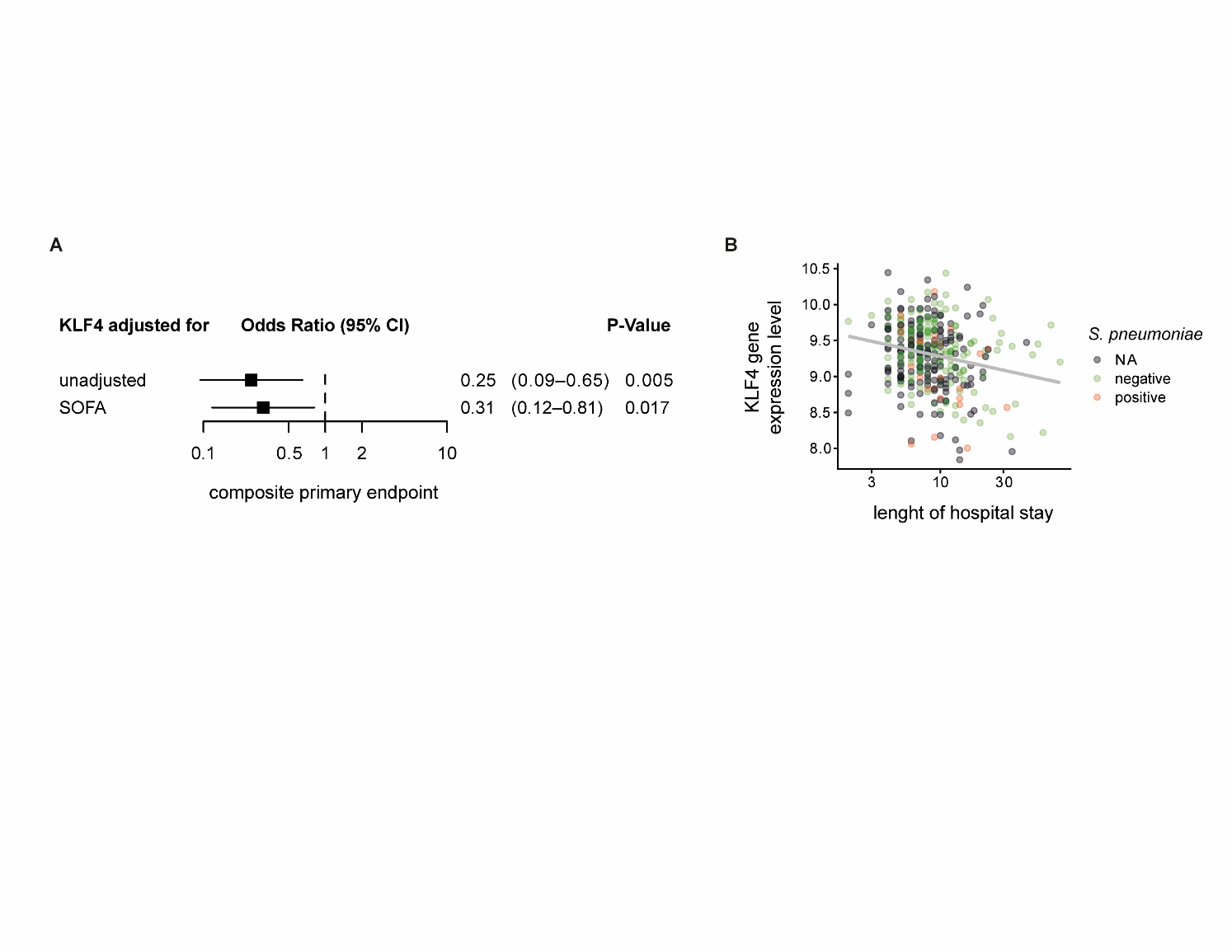


**Supplementary Figure S5: Correlation of KLF4 gene expression levels in peripheral blood with composite primary endpoint (death or intensive care unit within 28 days after admission) and length of hospital stay.** Correlation of KLF4 gene expression levels in peripheral blood of 371 patients with community-acquired pneumonia (CAP) at presentation to hospital with future composite primary endpoint (death or intensive care unit within 28 days after admission) (A) and length of hospital stay (B). Composite primary endpoint results were not adjusted or adjusted to SOFA score upon hospital admission (A). Green dots represent patients with positive testing for *S. pneumoniae* (blood culture, culture of respiratory material, or urinary antigen testing), red dots represent patients with negative testing for *S. pneumoniae*, and grey dots represent patients without testing for *S. pneumoniae* (NA) (B). Graphs show forest plot of two odds ratios (correlation unadjusted or adjusted to initial SOFA score) and associated confidence intervals (95% confidence level) (A), or scatter plot with trendline (B). Statistics: logistic regression (KLF4 gene expression level = explanatory variable; composite primary endpoint = outcome variable) (A), or Spearman’s rank-order correlation (correlation coefficient rho = -0.21, p-value = 4.52e-05) (B).

**Supplementary Table 1:** **demographic and clinical characteristics of the patient cohort.** CRB-65, clinical severity score for community-acquired pneumonia (confusion, respiratory rate ≥ 30/min, blood pressure systolic < 90 mmHg or diastolic ≤ 60 mmHg, age ≥ 65 years); CURB-65, clinical severity score for community-acquired pneumonia (confusion, urea blood concentration, respiratory rate ≥ 30/min, blood pressure systolic < 90 mmHg or diastolic ≤ 60 mmHg, age ≥ 65 years); PSI, pneumonia severity index; qSOFA, quick SOFA score.

|  | Overall (N=371) |
| --- | --- |
| Age, years | 59.1 (17.2) |
| Age ≥ 60 | 199 (53.6%) |
| Male sex | 220 (59.3%) |
| Nursery home resident | 8 (2.2%) |
| Smoking history | 232 (67.2%) |
| Chron. lung disease | 117 (31.7%) |
| Chron. cardiovascular disease | 104 (28.7%) |
| Chron. renal disease | 35 (9.6%) |
| Chron. liver disease | 35 (9.6%) |
| Chron. cerebrovascular disease | 22 (5.9%) |
| Tumor disease | 38 (10.3%) |
| Any of these comorbidities | 203 (55.5%) |
| Disoriented | 27 (7.3%) |
| Low blood pressure (Systolic < 90 mmHg or Diastolic ≤ 60 mmHg) | 165 (44.7%) |
| High respiratory rate (≥ 30/min) | 72 (22.9%) |
| Vasopressors | 16 (4.3%) |
| Mechanical ventilation within 28 days | 32 (13.0%) |
| ICU within 28 days | 85 (25.8%) |
| Length of hospital stay (days) | 10.3 (8.5) |
| In-hospital mortality | 12 (3.3%) |
| 28-day mortality | 15 (4.0%) |
| 90-day mortality | 21 (5.7%) |
| CRB-65 |  |
| 0 | 92 (24.9%) |
| 1 | 153 (41.5%) |
| 2 | 92 (24.9%) |
| 3 | 30 (8.1%) |
| 4 | 2 (0.5%) |
| Missing | 2 |
| CURB-65 |  |
| 0 | 80 (21.6%) |
| 1 | 106 (28.6%) |
| 2 | 100 (27.0%) |
| 3 | 60 (16.2%) |
| 4 | 23 (6.2%) |
| 5 | 2 (0.5%) |
| PSI |  |
| 1 | 77 (20.8%) |
| 2 | 90 (24.3%) |
| 3 | 73 (19.7%) |
| 4 | 89 (24.0%) |
| 5 | 42 (11.3%) |
| qSOFA |  |
| 0 | 118 (31.8%) |
| 1 | 190 (51.2%) |
| 2 | 57 (15.4%) |
| 3 | 6 (1.6%) |
| SIRS |  |
| 1 | 26 (7.1%) |
| 2 | 307 (83.4%) |
| 3 | 35 (9.5%) |
| Missing | 3 |
| SOFA | 3.2 (2.5) |

**Supplementary Table 2: primer sequences for KLF4 genotyping**

| primer name | primer sequence (5’ to 3’) |
| --- | --- |
| Lyz olMR 3066 mutant | CCCAGAAATGCCAGATTACG |
| Lyz olMR 3067 common | CTTGGGCTGCCAGAATTTCTC |
| Lyz olMR 3068 WT | TTACAGTCGGCCAGGCTGAC |
| KLF4 exon 1 | CTGGGCCCACATTAATGA |
| KLF4 exon 2 | AGTCTGACATGGCTGTCAGCG |
| KLF4 intron | CAGAGCCGTTCTGCCTGTTTT |

**Supplementary Table 3: exclusion criteria for enrollment in the PROGRESS study.** HIV, human immunodeficiency virus infection; AIDS, acquired immunodeficiency syndrome; ALI, acute lung injury; ARDS, acute respiratory distress syndrome; NYHA, New York Heart Association classification.

| No written informed consent  Participation in the same study at an earlier date  Therapy restriction  Insufficient capacity in the clinical procedure  Hospitalization more than 48 hours ago  Pregnancy or breastfeeding  Home mechanical ventilation over a tracheostoma  Tumor disease with cytostatic therapy or radiotherapy within the last six months  Steroid therapy (> 20 mg Prednisolone equivalent per day since > 14 days before inclusion)  Non-steroidal immunosuppressive therapy within the last six months  Bone marrow transplantation  HIV/AIDS  Active tuberculosis  Poststenotic pneumonia with bronchial carcinoma  Acute pulmonary embolism  Massive aspiration  Extra-pulmonary caused ALI/ARDS or sepsis with extra-pulmonary focus  Heart insufficiency NYHA IV  Liver insufficiency Child-Pugh C  Cystic fibrosis |
| --- |
